# Using Phosphatidylinositol Phosphorylation as Markers for Hyperglycemic Related Breast Cancer

**DOI:** 10.3390/ijms21072320

**Published:** 2020-03-27

**Authors:** Nirupama Devanathan, Sandra Jones, Gursimran Kaur, Ann C. Kimble-Hill

**Affiliations:** 1 Department of Biology, Indiana University School of Medicine, Indianapolis, IN 46202, USA; ndevanat@iupui.edu; 2 Department of Biochemistry and Molecular Biology, Indiana University School of Medicine, Indianapolis, IN 46202, USA; sj74@iu.edu; 3 Department of Chemistry, Indiana University Bloomington, IN 47405, USA; gurskaur@iu.edu

**Keywords:** hyperglycemia, hormone receptor positive breast cancer, HER2 positive breast cancer, triple negative breast cancer, PI3K/AKT signaling

## Abstract

Studies have suggested that type 2 diabetes (T2D) is associated with a higher incidence of breast cancer and related mortality rates. T2D postmenopausal women have an ~20% increased chance of developing breast cancer, and women with T2D and breast cancer have a 50% increase in mortality compared to breast cancer patients without diabetes. This correlation has been attributed to the general activation of insulin receptor signaling, glucose metabolism, phosphatidylinositol (PI) kinases, and growth pathways. Furthermore, the presence of breast cancer specific PI kinase and/or phosphatase mutations enhance metastatic breast cancer phenotypes. We hypothesized that each of the breast cancer subtypes may have characteristic PI phosphorylation profiles that are changed in T2D conditions. Therefore, we sought to characterize the PI phosphorylation when equilibrated in normal glycemic versus hyperglycemic serum conditions. Our results suggest that hyperglycemia leads to: 1) A reduction in PI3P and PIP3, with increased PI4P that is later converted to PI(3,4)P2 at the cell surface in hormone receptor positive breast cancer; 2) a reduction in PI3P and PI4P with increased PIP3 surface expression in human epidermal growth factor receptor 2-positive (HER2+) breast cancer; and 3) an increase in di- and tri-phosphorylated PIs due to turnover of PI3P in triple negative breast cancer. This study begins to describe some of the crucial changes in PIs that play a role in T2D related breast cancer incidence and metastasis.

## 1. Introduction

In the United States, over 276,480 new female cases of breast cancer are expected in 2020 alone [1]. Nearly 80% of these cases are invasive ductal cancer (IDC) [2]. IDCs are molecularly subtyped as luminal (e.g., hormone responsive), human epidermal growth factor receptor 2-positive (HER2+), or basal-like (e.g., non-hormone). Though each of these IDC subtypes have distinct proliferative and invasive profiles, there are unifying trends in their glucose metabolism such as: (1) ~50% of cases across subtypes demonstrate perturbations in the insulin receptor signaling pathway during glucose metabolism and (2) type 2 diabetes (T2D) is a risk factor for the incidence of breast cancer and poorer outcomes [3,4,5,6,7,8]. T2D is characterized by insulin resistance and progressive beta cell dysfunction which leads to chronic hyperglycemia [9]. For example, women with T2D were reported to have a 15%–20% [10] higher risk of being diagnosed with breast cancer and experience 30%–60% greater mortality than the general population [10]. In understanding this phenomenon, several studies have proposed to define the pathophysiology of T2D, suggesting that T2D induces hyperinsulinemia which subsequently promotes estrogen receptor (ER) activity thereby promoting proliferation [11]. Interestingly, preliminary studies have suggested that T2D has the strongest association with luminal breast cancer subtypes, with an ER positive characterization [11]. Thus, greater ER activity promotes proliferative conditions in ER-dependent signaling pathways (i.e., insulin and estradiol) as well as downstream signaling pathways, such as PI3K/Akt in insulin, as well as subsequent growth pathways [11,12,13,14,15,16].

A survey of literature suggests that phosphatidylinositol (PI) signaling is a key area of focus when analyzing the links between hyperglycemia, T2D, and the onset of breast cancer. Therefore, we suggest a possible role for PI phosphorylation as a marker for specific breast cancer phenotypes linked to T2D related onset. Cancer microenvironment growth factors increase glucose metabolism, generally thought of as being important for increased growth, migration, and proliferation rates [17,18,19]. For each IDC breast cancer subtype, the distinct molecular characteristics have shown varying enzymatic expression of the 3′, 4′, and/or 5′ kinase or the opposing phosphatases (Table 1)**.** This suggests each cancer subtype should have specificity in their PI phosphorylation profile (i.e., relative expression levels of PI3P; PI4P; PI5P; PI(3,4)P2; PI(4,5)P2; PI(3,5)P2; PIP3) thus playing a role in their respective incidence rates and aggressiveness. Furthermore, several reports have shown a potential linkage between kinase and phosphatase mutations and enhanced/metastatic breast cancer phenotypes through changes in activity for cell adhesion proteins, delocalization of polarity lipids and proteins, and activation of invasion/migration signaling [20,21,22,23,24,25,26,27].

Site-specific phosphorylation of PIs has been shown to regulate and stabilize the formation of local membrane heterogeneities thought to play a role in normal membrane protein signaling and cellular adhesion complex formation [28,29,30,31]. We have previously found that non-phosphorylated PIs can drive the formation of local membrane heterogeneities [32]. Taken together, it suggests a significant role for changing expression levels of the different PI phosphorylation species in the dysregulation of normal cell–cell contacts, cell polarization, signal transduction, and membrane trafficking events [4,6,7,33,34]. While it is understood that many factors play a role in this T2D-cancer nexus (e.g., pharmaceutical treatments [9,13,33,35], hyperinsulinemia [3,12], and inflammation [5,36,37,38,39]), there is significant evidence that increased glucose metabolism, and therefore a hyperglycemic microenvironment, is fundamental in the development of cancer [12,40]. Hence, we endeavored to determine the T2D glycemic effects on the cancerous microenvironment by studying the related changes in the PI phosphorylation profile. In this study, we used fixed cell staining and cellular extraction spot blotting to analyze cancer type specific changes in the PI phosphorylation profile of hormone receptor positive (HR+), HER2+, and triple negative breast cancer (TNBC) cells as a function of serum glucose concentrations.

There has been consistent effort to understand the role of mutations and changing expression levels of PI kinases and phosphatases, extending our knowledge of protein signaling. However, these enzymes have specific roles in maintaining the cellular lipidome prompting others to characterize the PIs, generally by their fatty acyl chain content. Dória et al. (2012) has shown that cancerous cell lines have higher levels of certain PIs in comparison to all other lipids [55]. Kim et al. (2016) furthered our understanding by using several mammary cell lines to show a 3-fold increase of PI in PR+ metastatic cancer, and a ~4–6 fold increase in PI metastatic TNBC [56]. In addition to work done in cell lines, Yang et al. (2015) used normal and breast cancer patient plasma to show that PIs could be used to differentiate between healthy and cancerous mammary tissue, as well as characterize aggressiveness of the tumor [57]. Kawashima et al. (2013) and Cífková et al. (2014) also added to the field by using patient tissues to highlight that the PI lipidome was significantly different in cancerous tissues, suggesting certain PIs are linked to tumor growth [58] and invasion [59]. Other studies have also shown that PI expression levels are important makers for tumorigenesis and aggressiveness in prostate [60] and brain [61] cancers. These and other studies use fatty acyl chains to highlight the potential use of PIs as breast cancer biomarkers. However, few studies point to the diversity of the PI head groups in relationship to these acyl chains. The work presented here attempts to enhance the field by defining the various changes in PI head group phosphorylation in light of cancer specific changes in enzymatic activity.

## 2. Results

### 2.1. Hormone Positive Breast Cancer PI Trends

**Figure 1:**
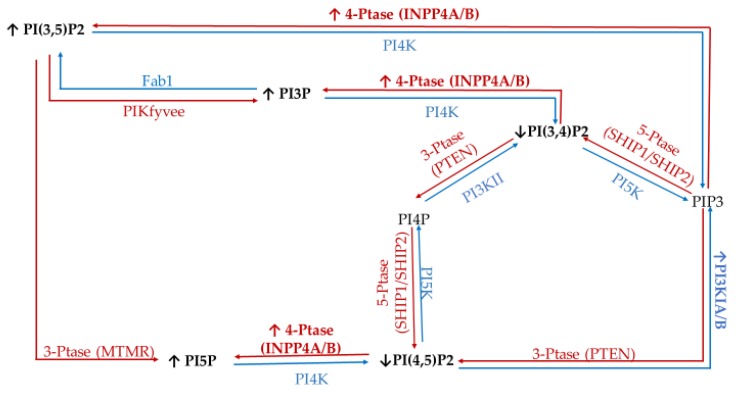
Estrogen receptor (ER+)/progesterone receptor (PR+) related expression of phosphatidylinositol (PI) kinases and phosphatases. Graphical representation of the hormone responsive breast cancer specific overexpression/increased activity (↑) and decreased expression/activity (↓) of the kinases (blue), phosphatases (red), as well as the hypothesized effects on the resulting lipidome.

To study the effect of hyperglycemia on hormone positive breast cancer, we utilized the estrogen receptor (ER+)/progesterone receptor (PR+) MCF7 breast cancer cell line. This metastatic cell line has several subtype specific characteristic changes in PI related enzymatic activity, namely in 3′-kinase Class I PI3K and 4′-phosphatase INPP4B. This cell line expresses a mutation in the Class I PI3K catalytic subunit which leads to an increase in activity linked to unregulated growth signaling [62,63,64,65,66,67]. This overexpression suggests an increase in the accumulation of PIP3 thought to be generated from PI(4,5)P2. Similarly, these ER+/PR+ cells lines typically express higher than normal levels of the 4′ PI phosphatase INPP4B [68]. The direct consequence of this higher than normal levels of INPP4B likely leads to the increased accumulation of PI3P, PI5P, and PI(3,5)P2 presumably generated from PI(3,4)P2, PI(4,5)P2, and PIP3, respectively. The net effect of these enzymatic changes for hormone positive breast cancer PI profile should be an increased PI3P, PI5P and PI(3,5)P2 content, and decreased PI(3,4)P2 and PI(4,5)P2 content (Figure 1).

#### 2.1.1. ER+/PR+ Breast Cancer PI Trends from Lipid Spot Blot Analysis

**Figure 2:**
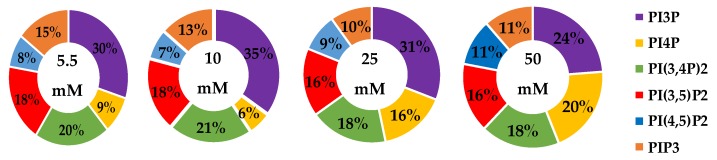
Effect of Glucose Concentration on ER+/PR+ breast cancer total PI phosphorylation. The % of total PI lipid PI3P, PI4P, PI(3,4)P2, PI(4,5)P2, PI(3,5)P2, and PIP3 from MCF7s incubated in 5.5 mM, 10 mM, 25 mM, and 50 mM serum glucose concentrations, measured by spot blot analysis (*n* = 3).

The whole cell lipidome percentage of each PI phosphorylation category in the MCF7 cell line across increasing glucose serum concentrations were obtained from the spot blots and summarized pictorially in Figure 2, while the average percentage of total lipid, standard deviation, and fold changes are presented in Table 2. The largest component of this profile was PI3P followed by PI(3,4)P2 and PI(3,5)P2 regardless of the serum glucose concentration. As predicted, the PI(4,5)P2 and PIP3 were the smallest components of the profile regardless of the serum glucose concentration. There were no statistically significant changes (*p* < 0.05) in the PI3P, di-phosphorylated, and PIP3 relative concentrations as a function of glucose serum concentration. There was also a statistically significant two-fold increase in the percentage of PI4P between 5.5 mM and 50 mM glucose serum concentrations (*p* < 0.05). In summary, these results suggest that hyperglycemia causes an overall depression of 4′-phosphatase activity in hormone responsive breast cancers.

#### 2.1.2. ER+/PR+ Breast Cancer PI Trends from Fixed Cell Fluorescence Analysis

**Figure 3:**
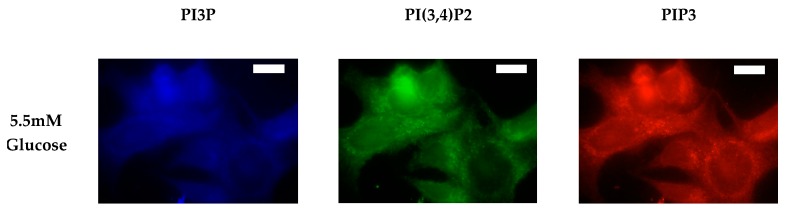

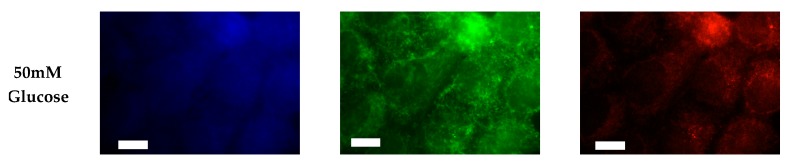
Glucose concentration changes cellular expression of PIPs in hormone receptor positive cells. Representative fixed cell fluorescent images of MCF7 cells cultured in normal (5.5 mM) and hyperglycemic (50 mM) serum glucose with immunofluorescent tags against PI3P, PI(3,4)P2, and PIP3. Scale bars represent 10 μm.

After characterizing the whole cell PI phosphorylation lipidome, we further endeavored to characterize the hormone responsive breast cancer cell line surface. Representative images demonstrating the comparative response between normal and hyperglycemic conditions of glucose serum concentrations in MCF7s across specific PIs are shown in Figure 3.

**Figure 4:**
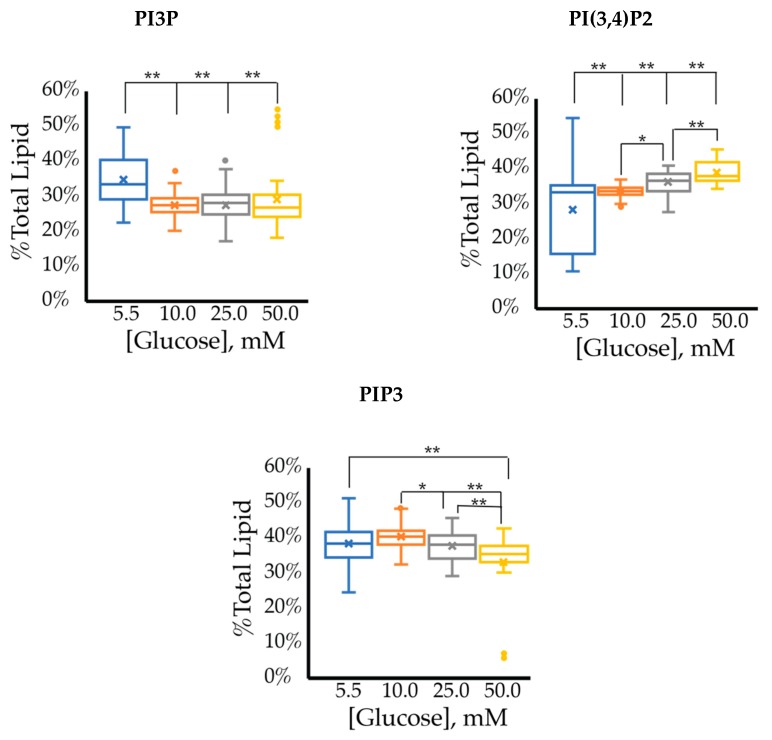
Effect of glucose concentration on MCF7 cell surface PI phosphorylation. PI3P, PI(3,4)P2, and PIP3 relative concentrations for MCF7s as determined by fluorescence intensity in fixed cells (*n* > 60). The % of total PI lipid for each mono-, di-, and tri-phosphorylated PI was determined through intensity analysis for the MCF7 cell line, incubated at 5.5 (**☐, blue**), 10 (**☐, orange**), 25 (**☐, grey**), and 50 (**☐, yellow**) mM serum glucose concentrations, respectively. * denotes *p* < 0.05 and ** denotes *p* < 0.005 as determined by a one-way ANOVA pair-wise comparison.

The cell surface expression of selected PI phosphorylation in the MCF7 cell line across increased glucose serum concentrations was obtained as fluorescent intensities from fixed cell surfaces (Figure 4). Overall, this assay suggested that the cell surface of hormone responsive breast cancers normally has PIP3 << PIP2 << PI3P in their relative concentrations (Table 3). In this assay, we observed a similar reduction in the amount of PI3P as a function of serum glucose concentration as in the whole cell analysis. However, the fixed cells suggested a very different trend for the di- and tri-phosphorylated PI profile. Specifically, a statistically significant increase was found in the amount of PI(3,4)P2 between 5.5 mM and 50 mM glucose serum concentrations. We also observed a small but statistically significant decrease in the amount of PIP3 between 10 mM and 50 mM glucose serum concentrations. In summary, these results showed that hyperglycemia caused cell surface expression of di-phosphorylated PIs to become the most dominant component, thereby suggesting an increase in hormone responsive breast cancers 4′-kinase and/or 5′-phosphatase activity.

Overall, these findings suggest that hyperglycemia led to a greater production of the whole cell PI4P while maintaining the di-phosphorylated and tri-phosphorylated PI content. Furthermore, the results suggested that hyperglycemia caused a decrease in PI3P and PIP3 and a greater production of cell surface PI(3,4)P2. PI4P is generally in the Golgi apparatus and a reasonable assumption would be that it was phosphorylated by PI3K upon transportation to the cell surface thereby generating increased amounts of cell surface PI(3,4)P2. However, this study also suggested that hyperglycemia leads to decreased relative amounts of PI3P, most likely due to an increase in PI4K activity.

### 2.2. HER2+ Breast Cancer PI Trends

**Figure 5:**
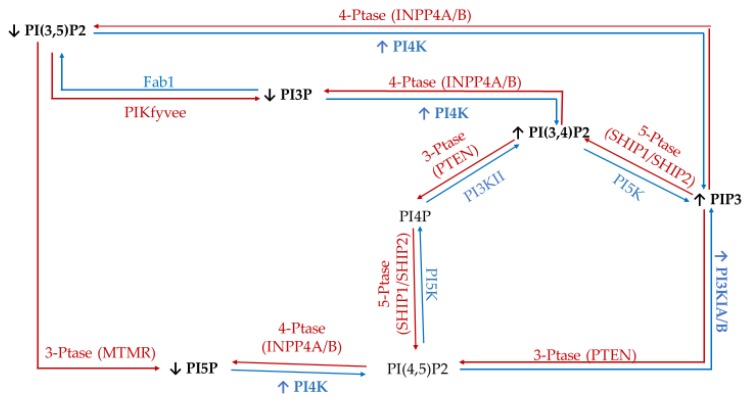
HER2+ related expression of PI kinases and phosphatases. Graphical representation of the cancer specific overexpression/increased activity (↑) and decreased expression/activity (↓) of the kinases (blue), phosphatases (red), as well as the hypothesized effects on the resulting lipidome have been noted.

To study the effect of hyperglycemia on HER2+ breast cancer, we utilized the metastatic SKBR3 cell line. The literature has identified HER2+ subtype specific aberrancies in PI phosphatases and kinases; namely PI3K Class I and PI4K [69,70]. Furthermore, the literature reports that cancer-associated mutations in the catalytic domain of PI3K increases the activity in HER2+ breast cancer [69,70]. This trend suggests an increase in PIP3 accumulation generated from PI(4,5)P2. Additionally, HER2+ breast cancer samples generally overexpress both PIP4K2B and PIP4K2A [71,72]. The overexpression of PI4K, therefore, suggests that there will be an increase in PI(4,5)P2 accumulation, generated from PI5P. This change may also lead to a decrease in PI(3,5)P2 as it is also converted into PIP3. Currently, there has been no report of changes in activity levels for the either the 3′ or 4′-phosphatases nor 5′-kinases/phosphatases. Therefore, we expect to see little to no change in the abundance of the mono and di-phosphorylated PI species. While there is little to inform predictions on the mono-phosphorylated PI lipids, action taken by PI3K and PI4K will act to both reduce and increase the relative pool of PI(4,5)P2, respectively, thus leading to no net change of this PIP2. In summary, HER2+ breast cancer should have an increased accumulation of PI(3,4)P2 and PIP3, while decreasing the overall PI(3,5)P2 content (Figure 5).

#### 2.2.1. HER2+ Breast Cancer PI Trends from Lipid Spot Blot Analysis

**Figure 6:**
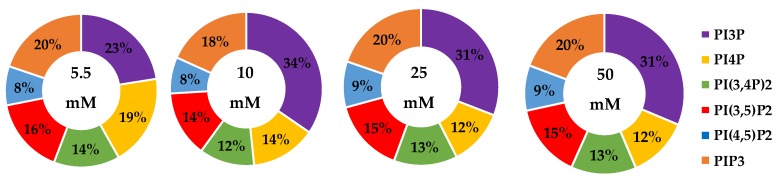
Effect of glucose concentration on HER2+ breast cancer total PI phosphorylation. The % of total PI lipid PI3P, PI4P, PI(3,4)P2, PI(4,5)P2, PI(3,5)P2, and PIP3 from SKBR3 cells incubated in 5.5 mM, 10 mM, 25 mM, and 50 mM serum glucose concentrations, measured by spot blot analysis (*n* = 3).

We analyzed the whole cell percentage of each PI phosphorylation in the SKBR3 cell line across increase glucose serum concentrations (Figure 6), while the average % total lipid, standard deviation, and fold changes are presented in Table 4. Overall, we found that regardless of glucose concentration, PI3P followed by PIP3 were most expressed in this cell line. While not statistically significant, PI3P appears to have a small increase proportional to the glucose serum concentration. We also found that PI(4,5)P2 was consistently the least expressed lipid. We observed a statistically significant decrease of PI4P lipid proportional to glucose serum concentration. No other lipid showed statistically significant response to serum glucose levels. Based on the enzymatic profile of this cancer, we hypothesized that the highest concentration components would be PI(3,4)P2 and PIP3, while PI(3,5)P2 and PI3P would have the lowest concentrations. While this hypothesis proved true for PIP3, the PI(3,4)P2 and PI(3,5)P2 had similar concentrations to one another at all glucose concentrations. Furthermore, PI3P concentrations were contrary to the hypothesis, with hyperglycemia causing an increase in its relative concentration. These results suggest that hyperglycemia impacts the ratio of PI3P to PI4P, probably by depressing of 4′-kinase and/or activating PTEN activity, within the HER2+ breast cancer cell.

#### 2.2.2. HER2+ Breast Cancer PI Trends from Fixed Cell Fluorescence Analysis

**Figure 7:**
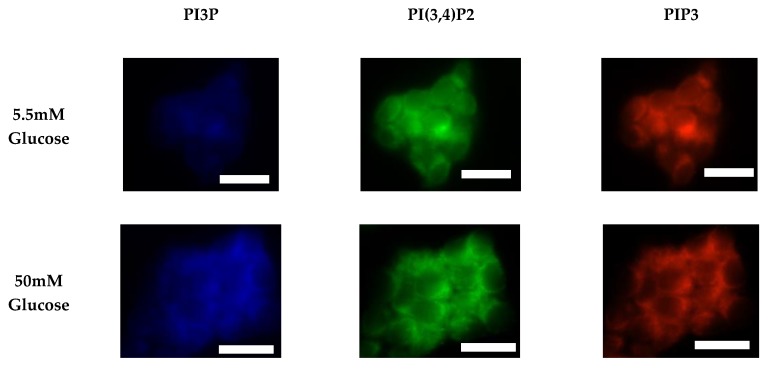
Glucose concentration changes cellular expression of PIPs in HER2+ cells. Representative fixed cell fluorescent images of SKBR3 cells cultured in normal (5.5 mM) and hyperglycemic (50 mM) serum glucose with immunofluorescent tags against PI3P, PI(3,4)P2, and PIP3. Scale bars represent 30 μm.

Characterization of the HER2+ whole cell PI phosphorylation lipidome was again followed with the characterization of the surface expression lipidome. Representative images demonstrating the comparative response between normal and hyperglycemic conditions of glucose serum concentrations in SKBR3s across specific PIs are shown in Figure 7.

**Figure 8:**
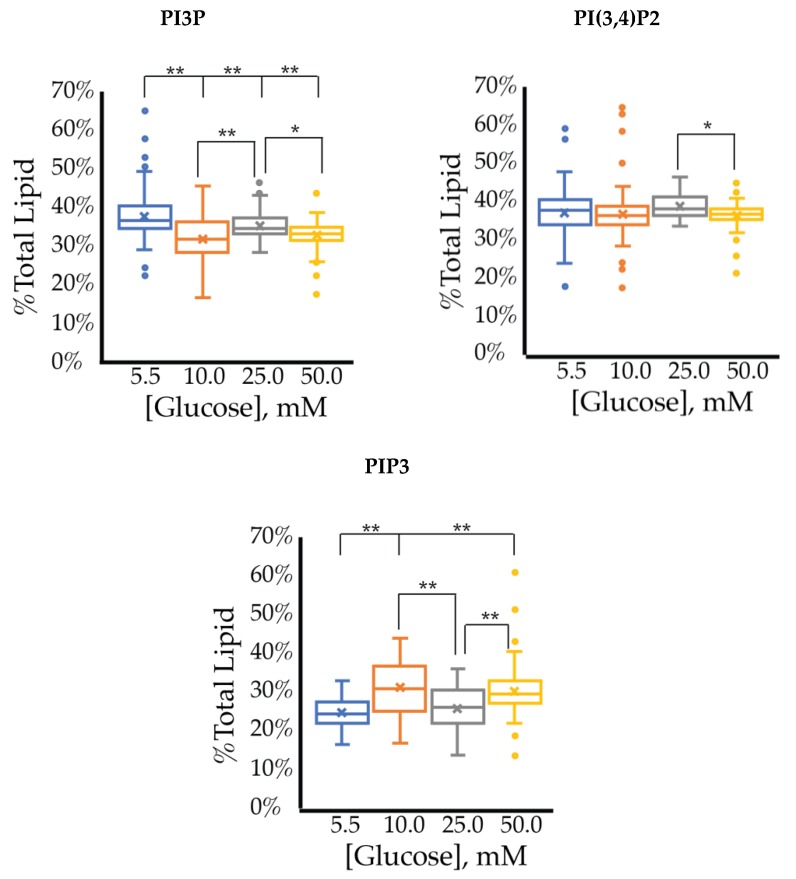
Effect of glucose concentration on PI3P, PI(3,4)P2, and PIP3 concentration across SKBR3 cell line (fluorescence intensity in fixed cells, *n* > 60). The % of total PI lipid for each mono-, di-, and tri-phosphorylated PI was determined through intensity analysis for the SKBR3 cell line, incubated at 5.5 (**☐**), 10 (**☐**), 25 (**☐**), and 50 (**☐**) mM serum glucose concentrations, respectively. * denotes *p* < 0.05 and ** denotes *p* < 0.005 as determined by a one-way ANOVA pair-wise comparison.

The cell surface expression of selected PI phosphorylation in the SKBR3 cell line across increased glucose serum concentrations are summarized in Figure 8. Overall, we observed that PI3P and PI(3,4)P2 are the major component of the profile irrespective of glucose serum concentration. However, unlike the whole cell assay, this experiment suggested a small decrease in the surface expression of PI3P as a function of serum glucose concentration. The relative concentration of PI(3,4)P2 had no statistically significant changes as a function of serum glucose concentration (Table 5). PIP3 showed a very small increase in surface expression as a function of serum glucose concentration (Table 5). Unlike the whole cell analysis, the cell surface was dominated equally with PI3P and PI(3,4)P2 suggesting that PI4K may have more of a localized effect in HER2+ breast cancer cells.

These findings were consistent with our hypotheses and suggested that hyperglycemia led to a greater overall production of PI3P, but a decrease in surface expression of PI3P. These findings could be due to the depressed activity of INPPB4 in this cell line. Furthermore, hyperglycemia led to a decrease in the PI4P which could be phosphorylated by hyperglycemic induced PI5K phosphorylation, followed by overactive PI3K phosphorylation to generate increasing amounts of PIP3 on the cell surface.

### 2.3. Triple Negative Breast Cancer (TNBC) PI Trends

**Figure 9:**
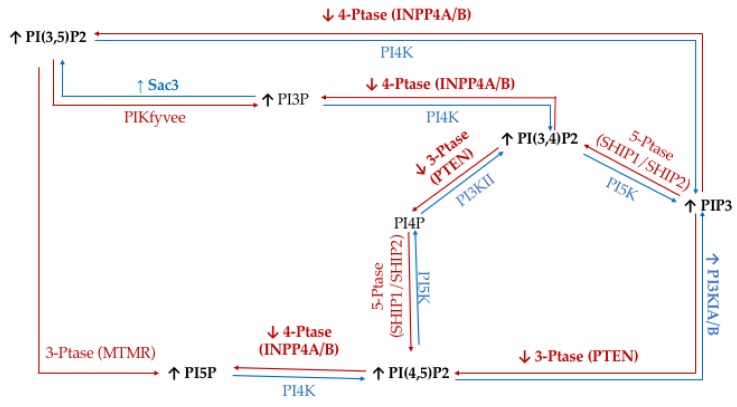
Triple Negative Breast Cancer (TNBC) related expression of PI kinases and phosphatases. Graphical representation of the cancer specific overexpression/increased activity (↑) and decreased expression/activity (↓) of the kinases (blue), phosphatases (red), as well as the hypothesized effects on the resulting lipidome have been noted.

To study the effect of hyperglycemia on TNBC, we utilized the metastatic MDA-MB-468 cell line. The literature has identified TNBC specific PI phosphatases and kinases aberrancies; namely PI3K, PTEN, and INPP4B. Through a metformin study, it was demonstrated that increasing serum glucose concentrations would increase the activity of glucose transporters and create lipid rafts in the cell surface of MDA-MB-468 cells [73]. Significantly, these lipid rafts have been demonstrated to clearly co-localize with several epidermal growth factor receptors (EGFRs), which is highly expressed in some of the most metastatic TNBCs [74,75]. EGFRs have been shown to affect downstream PI3K/Akt signaling through increased activity of the PI3K catalytic subunit alpha and reduced activity of the 3′-phosphatase PTEN and the 4′-phosphatase INPP4B [76,77]. In addition, it has been demonstrated that PTEN and INPP4B activity are lost in TNBC [78,79]. Furthermore, the 5′-phosphatase Sac3 expression levels are higher in TNBC, which has been linked to increased TNBC proliferation [53,80]. The net effect of these changes in enzymatic activity should be an accumulation of PI(3,4)P2, PI(3,5)P2, PI(4,5)P2, and PIP3 in TNBC (Figure 9).

#### 2.3.1. Triple Negative Breast Cancer PI Trends from Lipid Spot Blot Analysis

**Figure 10:**
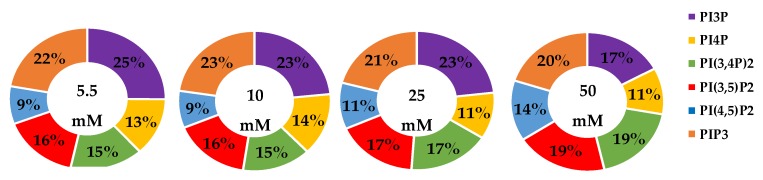
Effect of glucose concentration on TNBC breast cancer total PI phosphorylation. The % of total PI lipid PI3P, PI4P, PI(3,4)P2, PI(4,5)P2, PI(3,5)P2, and PIP3 from MDA-MB-468 cells incubated in 5.5 mM, 10 mM, 25 mM, and 50 mM serum glucose concentrations, measured by spot blot analysis (*n* = 3).

We analyzed the whole cell percentage of each PI phosphorylation in the MDA-MB-468 cell line across increasing glucose serum concentrations (Figure 10), while the average % total lipid, standard deviation, and fold changes are presented in Table 6. At normal glucose serum conditions, the lipidome was largely dominated by PI3P and PIP3. The next largest components were almost equally distributed between PI4P, PI(3,4)P2, and PI(3,5)P2. Similar to all of the other breast cancer cell types studied, the smallest component on the whole cell lipidome was PI(4,5)P2. In general, hyperglycemia appears to have led to decreased PI3P and increased di-phosphorylated PI lipids. However, due to the spread of the data there was no statistically significant change in the PI lipidome as a function of serum glucose concentration. Based on the previously discussed metformin studies in TNBC, we had hypothesized that hyperglycemia would lead to a significant increase in di- and tri-phosphorylated PI content. However, these data suggested that this phenomenon was not occurring globally with statistical significance within these cells. Hence, these results suggested that hyperglycemia alone cannot trigger the PI3K, PTEN, and INPP4B impacts seen in the metformin study, while potentially muting the TNBC PI3K and Sac3 activity.

#### 2.3.2. Triple Negative Breast Cancer PI Trends from Fixed Cell Fluorescence Analysis

**Figure 11:**
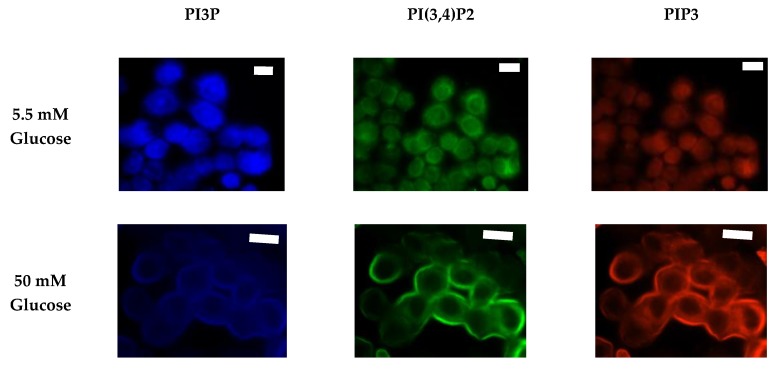
Glucose concentration changes cellular expression of PIPs in TNBC cells. Representative fixed cell fluorescent images of MDA-MB-468 cells cultured in normal (5.5 mM) and hyperglycemic (50 mM) serum glucose with immunofluorescent tags against PI3P, PI(3,4)P2, and PIP3. Scale bars represent 30 μm.

We again followed characterization of the whole cell PI phosphorylation lipidome with the characterization of the MDA-MB-468 surface expression. Representative images demonstrating the comparative response between normal and hyperglycemic conditions of glucose serum concentrations in MDA-MB-468s across specific PIs are shown in Figure 11.

**Figure 12:**
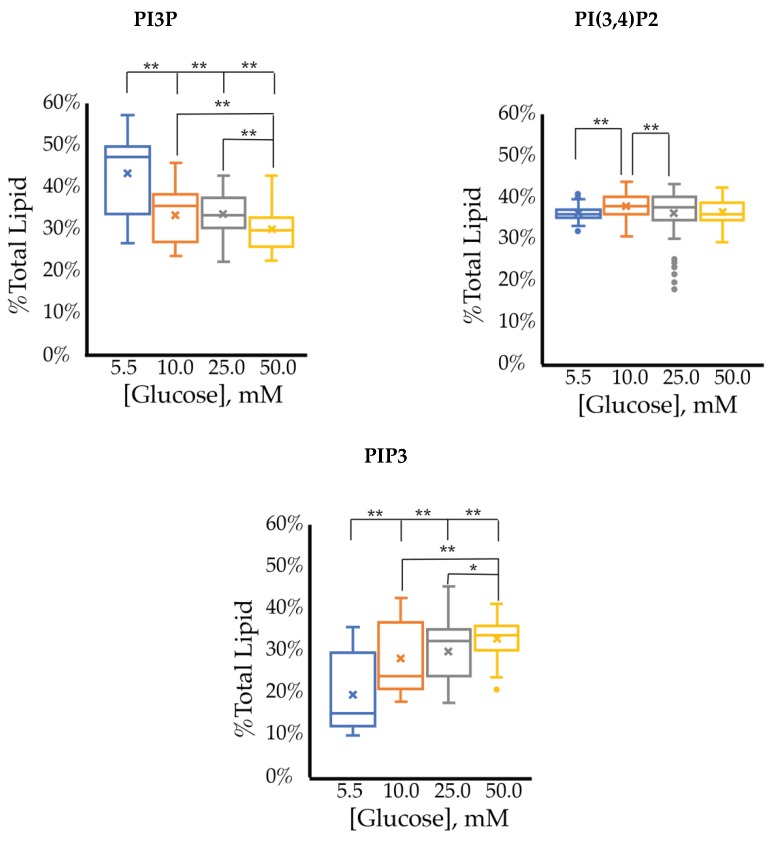
Effect of glucose concentration on PI3P, PI(3,4)P2, and PIP3 concentration across MDA-MB-468 cell line (fluorescence intensity in fixed cells, *n* > 60). The % of total PI lipid for each mono-, di-, and tri-phosphorylated PI was determined through intensity analysis for the MDA-MB-468 cell line, incubated at 5.5 (**☐**), 10 (**☐**), 25 (**☐**), and 50 (**☐**) mM serum glucose concentrations, respectively. * denotes *p* < 0.05 and ** denotes *p* < 0.005 as determined by a one-way ANOVA pair-wise comparison.

The cell surface expression of selected PI phosphorylation across increased glucose serum concentrations are summarized in Figure 12 with analysis of those changes in Table 7. At normal glucose conditions, the cell surface was dominated by PI3P followed by PI(3,4)P2 content. The cell surface exhibited a much lower PIP3 content than in the whole cell lipidome. As the glucose concentration increased, there was a statistically significant dose response where PI3P was decreased (−1.6 ± 0.1% total PI/mM) while PIP3 was increased (1.9 ± 0.3% total PI/mM). This PI3P result was similar but much more statistically significant than that of the whole cell lipidome, while this PIP3 effect was not seen in the whole cell lipidome. There was also a statistically significant increase in PI(3,4)P2 content, but the rate of change was small in comparison to the other two components. Therefore, the hyperglycemia condition caused PI(3,4)P2 to be the largest component, followed equally by PI3P and PIP3. Unlike the whole cell, the cell surface lipidome reflected the expected net PI(3,4)P2 and PIP3 effects as hypothesized for TNBC. However, the similarity between the dose responses for PI3P and PIP3 suggested their interconversion may be a direct influence of hyperglycemia on the depression of INPP4B activity and increased expression of Sac3 as previously discussed.

Overall, these findings suggest that TNBC in normal glucose conditions can largely be characterized by its PI3P content and that hyperglycemia leads to increased di- and tri-phosphorylated PIs due to a turnover of PI3P and decreased turnover of PIP3.

### 2.4. Comparison of Cell Lines

**Figure 13:**
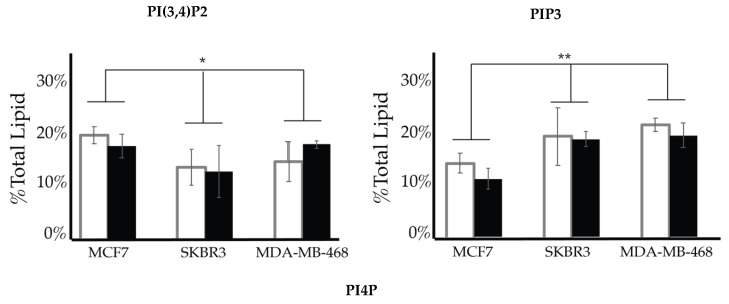

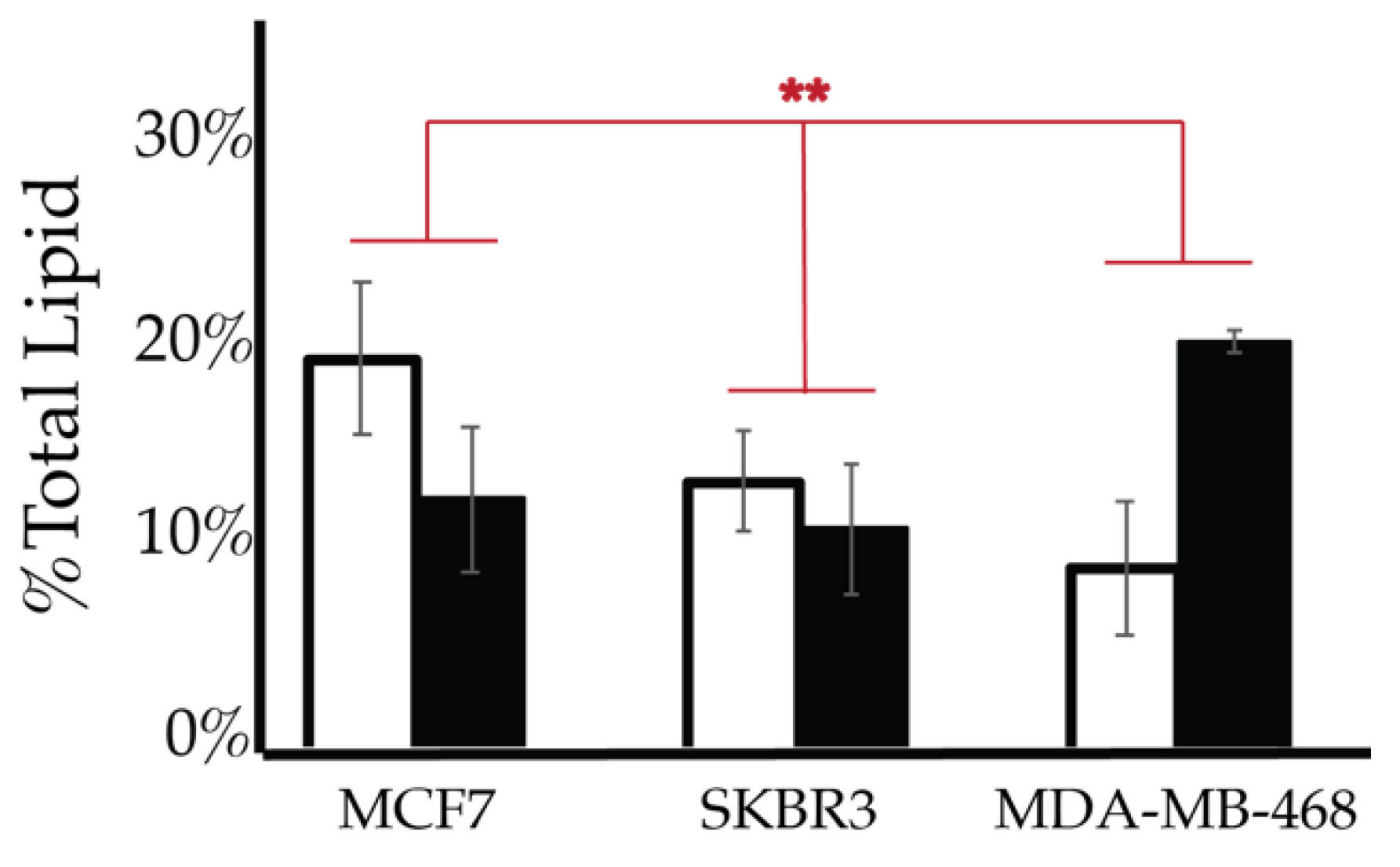
Comparison of whole cell lipidomes across cell types. Comparison of the PI(3,4)P2 and PIP3 content of each of the studied cell lines where the normal (❒) and hyperglycemic (∎) serum conditions led to two-way ANOVA statistically significant differences (* denotes *p* < 0.05, ** denotes *p* < 0.005). Additionally, PI4P content had a two-way ANOVA statistically significant interaction effect across cell types suggesting another variable influences their relationship (** denotes *p* < 0.005).

After characterizing the PI lipidome of these three types of cancerous cell lines, we further endeavored to find characteristic differences between them. We performed a 2-way ANOVA comparison of the MCF7, SKBR3, and MDA-MB-468 whole cell lipid components to find differences in their profiles. As a result, we have found that PI(3,4)P2 and PIP3 show statistically significant differences in their whole cell expression profiles (Figure 13). While PI4P showed no statically significant differences in the cell types of serum conditions, the 2-way ANOVA comparison did suggest that there was another factor that played a role in the difference in its expression that may be explained by the enzymatic differences previously discussed. Upon further analysis of the PI(3,4)P2 content in these three cell lines at normal glycemic conditions, we found that MCF7′s higher content than that found in SKBR3 is the most pronounced difference (1-way ANOVA statistical analysis, *p* < 0.05). However, there were no differences in the PI(3,4)P2 content for the three cell lines when in hyperglycemic conditions. PIP3 content at normal glycemic conditions showed that MCF7s had a statistically significant lower content than that of MDA-MB-468s (1-way ANOVA statistical analysis, *p* < 0.05). In this case, MCF7 PIP3 content at hyperglycemic conditions was also statistically different between it and SKBR3s and MDA-MB-468s.

**Figure 14:**
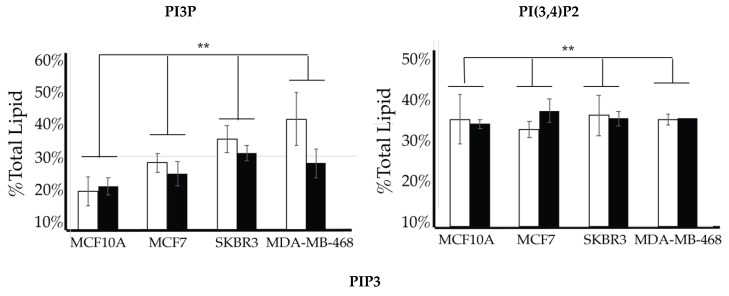

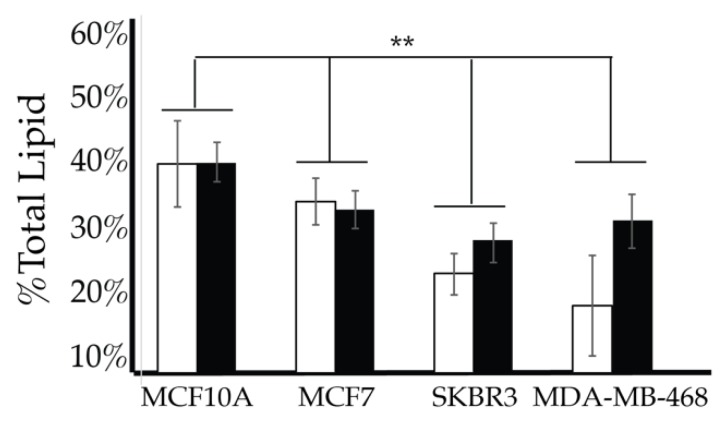
Comparison of cell surface lipidomes across cell types. Comparison of the PI3P, PI(3,4)P2, and PIP3 content of each of the studied cell lines where the normal (❒) and hyperglycemic (∎) serum conditions led to two-way ANOVA statistically significant differences (** denotes *p* < 0.005).

We then looked for characteristic differences between the cell surface expression of across all cell lines. The 2-way ANOVA comparison of the MCF10A, MCF7, SKBR3, and MDA-MB-468 cell surface components that PI3P, PI(3,4)P2 and PIP3 all show statistically significant differences (*p* < 0.005) in their profiles (Figure 14). Statistically, each of the cancers are different in their cell surface compositions as well as their amount of change between glycemic conditions compared to the non-cancerous breast cell line (*p* < 0.005). An additional comparison between MCF7, SKBR3, and MDA-MB-468 cells surface composition suggests that each cancer type is also statically different (*p* < 0.05). These results suggested that at normal glycemic conditions, hormone responsive breast cancers had a characteristically low PI3P/high PIP3 content while TNBC had a characteristically high PI3P/low PIP3 content. Hyperglycemic conditions also led to significant differences in their cell type specific profiles. Thus, we suggested that hyperglycemia led to hormone responsive breast cancer low PI3P/high PI(3,4)P2/high PIP3 content and HER2+ breast cancer high PI3P/low PIP3 content.

Our approach to lipidomic changes in hyperglycemia not only models changes seen between cancer types, it also distinguishes neoplastic cells from non-cancerous mammary cell lines. As a proof of concept, the inclusion of data from the non-cancerous MCF10A cell line shows a grossly different lipid profile than its neoplastic counterparts, and the expression profile does not significantly change as a function of serum glucose concentration. The results shown here suggest the need for robust follow-up studies to determine the role of 5′ phosphorylation in glucose sensing. Furthermore, this work suggests a need to expand our examination to other mammary cell lines, including the determination of all PI surface expression. This level of characterization may highlight lipidome differences related to ethnic health disparities and tumor aggressiveness. Hence, we suggest that this novel approach of using the PI lipidome to characterize and anticipate tumor behavior will have significant clinical implications.

## 3. Discussion

This study aimed to characterize the changes in the PI phosphorylation lipidomic profiles in response to glucose serum conditions in the different invasive ductal breast cancer subtypes. The results of this study indicate critical PI lipidomic changes in response to cellular reactions under increasing serum glucose concentrations. We demonstrated that a phosphorylation lipidomics approach better informs the mechanisms under which hyperglycemia can induce both proliferative and metastatic character, distinguishing even between the subtypes of breast cancer. At the same time, the findings from this study demonstrate that despite known aberrancies to the phosphatases and kinases that govern the interconversion of PIs, the lipidomic changes that result do not align entirely. This observation underscores the importance of analyzing lipidomic profiles and the accumulation of specific lipids in addition to the proteomic and genomic approaches generally used.

Many of these PI lipids play key roles in not only maintaining the cellular membrane polarity but also the trafficking and stabilizing of proteins key in mammary tumorigenesis and metastasis (Table 8). While PI3K/Akt signaling is one critical nexus between T2D and breast cancer that has been shown to play an important role in glucose metabolism through glycolysis mediated via cytoskeletal remodeling [44], assessing the changes in the accumulation of 4′- and 5′-phosphorylation in response to changing serum glucose concentrations has illustrated a potential breast cancer specific role for their related kinases and phosphatases.

In the hormone receptor positive cancers, we discovered that PI4P and PI(3,4)P2 best demonstrated potential as markers for changes related to hyperglycemic serum conditions. PI4P has been shown to impair Golgi-endosomal trafficking and create a pro-tumorigenic environment via interactions with receptors implicated in tumorigenesis and downstream regulation of the migratory and proliferation pathways [51]. Though localized to the Golgi apparatus, PI4P promotes tumor motility, angiogenesis, invasion, and metastasis [51]. PI(3,4)P2 has also been implicated in metastasis as its upregulation leads to an increase in invadopodia formation and loss of focal adhesions [91]. These cells also showed a significant decrease in the cell surface expression of PI3P. PI3P plays a distinct role in regulating endocytosis where its loss is indicative of a loss of endocytosed resident and transcytosing apical membrane proteins localized to the plasma membrane, thereby signaling a loss of cellular polarity, similar to treatment with the PI3K inhibitor wortmannin [104]. Thus, an upregulation of PI4P and PI(3,4)P2, while downregulating PI3P, due to hyperglycemia suggests that these cells have developed less polarized and more invasive and migratory phenotypes. While these studies were done using the MCF7 cell line, a metastatic cell line originating from pleural effusion [105], the work is highly correlative with other studies that reported a higher T2D incidence, metastasis, and mortality rates of invasive estrogen receptor–positive breast cancer in post-menopausal women when the diabetes is untreated by metformin [35,106].

Alternatively, the trends from the HER2+ cell line demonstrated the exact opposite in regard to PI4P. Hyperglycemia led to a decrease in the PI4P, which we postulated to be due to hyperglycemic induced PI5K phosphorylation and overactivity of PI3K phosphorylation, thereby generating increasing amounts of PIP3 on the cell surface. This PI4P effect suggests that HER2+, unlike the hormone responsive breast cancer, cells undergo an increased Golgi-endosomal activity that should decrease migratory and proliferation pathways. However, PIP3 is generally localized to the basolateral membrane of polarized cells and an overabundance of this lipid causes the formation of apical membrane protrusions of over-accumulated basolateral proteins, a clear sign of a loss of polarity [27]. These types of protrusions have been thought to be key in cancer migration [40]. While these results were found in the SKBR3 cell line, a HER2+ cell line derived from the metastases in the pleural effusion [107], it suggests that hyperglycemia leads these cells to take on a more aggressive phenotype. Hyperglycemia in this cell line also led an increase in overall production of PI3P, but a decrease in surface expression of PI3P. Like the hormone responsive breast cancers, this decrease in PI3P localization at the cell surface would lead to a loss of cellular polarity. In total, this suggests that the hyperglycemic effect on HER2+ breast cancer cells will lead to the loss of normal mammary cell like shape and polarity, and begin the formation of more mesenchymal-like morphologies that are indicative of invasive cancer [108]. These results may shed light on why T2D has been shown to lead to decreased survival rate as HER2+ breast cancer advances [109]. Use of insulin (known to activate parallel signaling as the HER2 receptor) has been shown to decrease overall survival rate while use of metformin increases overall survival rates [109,110]. Furthermore, studies have shown that black patients with HER2+ breast cancer were twice as likely to have T2D and more likely to present with stage IV metastatic breast cancer within 12 months [111]. Therefore, this work shows a clinical need to further investigate the role of the PI lipidome in the HER2+/T2D nexus as well as a possible intersection for the racial disparities associated with this disease.

Finally, hyperglycemia in TNBC in led to a decisive increase in PIP3 due to phosphorylation of PI3P and the associated di-phosphorylated PIs. As discussed within the context of HER2+ breast cancer, hyperglycemic related increases in PIP3 leads to overall dysregulation of the cellular polarity causing extensions suggestive of transition to a more mesenchymal cell type. This would be further exasperated with the loss of the PI3P, which is known to lead to a loss of apical membrane proteins localized within the plasma membrane. While T2D has been linked to the onset and aggressiveness of the other breast cancers in this study, T2D premenopausal breast cancer patients are more often diagnosed with hormone receptor negative, HER2-negative, TNBC [10]. TNBC also presents more frequently in women of African descent or with BRCA mutations generally found in women of Ashkenazi Jewish descent [112,113,114,115,116]. TNBCs are generally seen as being more aggressive, have fewer treatment options, and present as higher grades than the other types. This work was done with MDA-MB-468 cells, which originate from the metastasis site in the pleural effusion of a black woman. Thus suggesting the lipidomic results in this report provide insight as to the cellular mechanisms that lead to T2D related TNBC aggression. However, future studies would also compare these results with those generated from a Caucasian originated sample to define the role in racial disparities in this breast cancer type.

Though the different breast cancer types may have similar proteomic level changes, i.e., the activating mutation or overexpression of PI3K across subtypes, a lipidomics approach demonstrates that each subtype can have distinct PI phosphorylation profiles that are drastically different upon the introduction of hyperglycemic conditioning. Even though it has been previously proposed that the mechanisms associated with hyperinsulinemia and inflammation act as an exclusive driver of breast cancer in T2D, our study suggests that through lipidomic remodeling hyperglycemia independently encourages tumorigenic processes. Rising glucose levels without a high insulin state were associated with the tumor’s acquisition of varying degrees of invasive and proliferative character in hormone receptor positive and TNBC cell lines. This finding elucidates the multi-modal effects of the diabetic state on breast cancer tumorigenesis. More significantly, however, our study considers an emerging role for lipid-based biomarkers to distinguish between subtypes of breast cancer; as a case in point, each cell line demonstrated a distinct lipidomics profile. Our results suggested that marker(s) for hyperglycemic related breast cancer should be: 1) reduction in PI3P and PIP3 with increased PI4P and PI(3,4)P2 in HR+; 2) reduction in PI3P and PI4P with increased PIP3 in HER2+; and 3) increased PIP3 with decreased PI3P in TNBC. 

## 4. Materials and Methods

### 4.1. Materials

Human breast tissue cell lines (MCF7, SKBR3, MDA-MB-468) were gifts from Dr. Clark Wells (Indiana University School of Medicine, Indianapolis, IN, USA). Newborn Calf Serum (NCS) was purchased from Peak Serum (Denver, CO, USA). Antibodies against PI3P (Z-P003), PI4P (Z-P004), PI(3,4)P2 (Z-P034b), PI(3,5)P2 (Z-P035), PI(4,5)P2 (Z-P045), and PIP3 (Z-P345) were obtained from Echelon Biosciences (Salt Lake City, UT, USA). All other materials were purchased from Fisher Scientific (Pittsburgh, PA, USA).

### 4.2. Methods

#### 4.2.1. Mammalian Cell Culture

Breast cancer cell lines were normally cultured in DMEM with 10% NCS at 37 °C in 5% CO_2_ (*v*/*v*) as previously described [117,118,119,120]. Cells were plated and at ~80% confluency, cells were rinsed twice in phosphate buffered saline solution, and then incubated for 24 h in no glucose DMEM, enriched with 10% *v/v* NCS and a 0.5 kg/L glucose stock solution for final solutions at 5.5 mM (normal), 10 mM (transition), and 25 and 50 mM (hyperglycemia) [121].

#### 4.2.2. Fixed Cell Imaging

Cells were rinsed twice with phosphate buffered saline solution (PBS). The cells were then fixed on the cover slips with 4% PFA in PBS, washed 3 times with PBS, permeabilized by 0.5% saponin, and again washed with tris buffered saline (TBS). The cells were blocked with 10% goat serum in TBS at 4 °C overnight followed by incubated for 1 hr at 37 °C with antibodies previously conjugated to Alexa Fluor 350, Alexa Fluor 488, and Alexa Fluor 594 (1:40,000 dilution) and washed 3 times with TBS [122]. The cells were rinsed thoroughly with diH_2_O, dried completely, and sealed with mounting media to slides. Imaging was done using Zeiss Axiovision (v. 48) on a Zeiss Axio Observer Z1 confocal microscope (Carl Zeiss Microscopy, Jena, Germany) using a 60× oil objective [117,118,119] where each cell line had consistent exposure times across the biological triplicates. PI3P, PI(3,4)P2, and PIP3 was observed using the 4′,6-diamidino-2-phenylindole (DAPI), green fluorescent protein, and Texas Red filter sets, respectively. Quantification of the fixed cell fluorescence intensity was performed through ImageJ v2.0.0 (National Institute of Mental Health, Bethesda, MD, USA). Fluorescence intensity measurements were obtained by recording the Integrated Density, which was used to determine the percentage of the total fluorescence between the three components. The analysis was performed using biological triplicates with *n* > 20 cells in each replicate.

#### 4.2.3. Spot Blot Assay

Cells were rinsed twice with phosphate buffered saline solution and then lysed in radioimmune precipitation assay buffer (50 mM Tris pH 8.0, 2 mM EDTA, 10% Triton X-100, 150 mM NaCl, 0.1% sodium dodecyl sulfate containing 1 mM NaVO_4_, 2 mM β-glycerol phosphate, and protease inhibitor mixture (Sigma-Aldrich, St. Louis, MO, USA). Lysates were blotted onto the Ambersham Hybond ECL membrane and dried. The membrane was then blocked at room temperature for 30 min in phosphate buffered saline buffer containing 0.1% Triton-X and 1% (*w*/*v*) nonfat dry milk. The membrane was then incubated for an additional hour with the specific PI primary antibody, washed in phosphate buffered saline with 0.1% Triton-X, and incubated with the secondary antibody fluorescently labeled with Alexa Fluor 790 (1/1000 dilution) (A11371, Thermo Scientific, Waltham, MA, USA). The membrane was rinsed in phosphate buffered saline with 0.1% Triton-X, and the bound protein was then analyzed using a LiCOR Biosciences imaging station and Odyssey v1.2 was used to quantify the fluorescence intensity previously reported [117,120].

#### 4.2.4. Statistical Analysis

The whole cell percentage of each PI phosphorylation in the MCF7, SKBR3, and MDA-MB-468 cell lines across increase glucose serum concentrations were analyzed with the two-tailed Student t-test, which assumed two sample equal variance using an alpha value of 0.05. The cell surface percentage expression of each PI phosphorylation in the MCF10A, MCF7, SKBR3, MDA-MB cell lines across increased glucose serum concentrations was compared to normal glycemic conditions using the One-Way ANOVA analysis. The Q-test was used as a post-hoc test to determine significance between each of the groups, where the alpha value was 0.05. Differences in the profiles of the whole cell lipid components of MCF7, SKBR3, and the MDA-MB-468 cell lines were determined using a Two-Way ANOVA was performed where the alpha value was 0.05. All statistical analysis was done using the Microsoft Excel Data Analysis Toolpak.

## Figures and Tables

**Table 1:** Phosphatidylinositol enzymes with known modulations of function in cancer

Enzyme Type	Enzyme	Target	Product	Modulation of Function Found in Specific Cancers
Kinases	Class I PI3Ks	PI	PI3P (disfavored in vivo)	Amplification in 10% of breast cancer cases (increased activity) [41] mutated in ~40% of all breast cancers [42].
		PI4P	PI3,4P (disfavored in vivo)	
		PI(4,5)P2	PIP3 (in vivo)	
	Class II and III PI3K (e.g., hVps34)	PI	PI3P	Involved in Golgi vesicular transport and lysosomes [43]. Produces endosomal PI3P. Promotes cell proliferation signaling [44].
		PI4P	PI(3,4)P2	
	type II/III family PI 4-kinases (PI4K)	PI	PI4P	Loss of PI4KIIα/β linked to triple negative breast cancer (TNBC) apoptosis [45]. PI4KIIIβ overexpression promotes proliferation signaling [46] and causes disruption of normal mammary duct formation [47].
	PI 5-kinase (PI5K)	PI4P	PI(4,5)P2	PIP5KI important for invadopodia formation [48]
	PIKfyve	PI	PI5P	Regulate the cell cycle via epidermal [49] growth factor receptor (EGFR), positive regulator of GLUT4 [28] trafficking.
		PI3P	PI(3,5)P2	
Phosphatases	Myotubularin family of phosphatases (e.g., MTMRs)	PI3P	PI	MTMR3 highly expressed in TNBC, promotes aggression [50].
	Sac1	PI4P	PI	Knock-down induces epithelial to mesenchymal cell transition [51].
	3′-phosphatases (e.g., PTEN)	PI(3,4)P2	PI4P	Mutated (loss of function) in late-stage tumors, tumor-suppressor [43,52,53].
		PI(3,5)P2	PI5P	
		PIP3	PI(4,5)P2	
	4′-phosphatases (e.g., INPP4A and INPP4B)	PI(3,4)P2	PI3P	Tumor suppressor in breast cancer [28].
		PI(4,5)P2	PI5P	
		PIP3	PI(3,5)P2	
	5′-phosphatases (e.g., Synaptojanin-2, SHIP, OCRL, Sac3) Class I PI3Ks	PI(3,5)P2	PI3P	Sac3 overexpressed in TNBC [53]. SHIP1/2 necessary for breast cancer invasion and metastasis [54]. Amplification in 10% of breast cancer cases (increased activity) [41] mutated in ~40% of all breast cancers [42].
		PI(4,5)P2	PI4P	
		PIP3	PI(3,4)P2	

**Table 2:** Statistical analysis of whole cell lipidome in MCF7 Cell Line.

Serum Glucose Concentration		PI3P	PI4P	PI(3,4)P2	PI(3,5)P2	PI(4,5)P2	PIP3
**5.5 mM**	Average %	30	9	20	19	8	15
	Std. Error	5	3	1	1	4	1
**10 mM**	Average %	35	6	21	18	7	14
	Std. Error	5	3	1	1	4	1
	Fold Change	1	1	1	1	1	1
**25 mM**	Average %	31	16 *	18	16	9	10
	Std. Error	5	3	1	1	4	1
	Fold Change	1	2	1	1	1	1
**50 mM**	Average %	24	20 *	18	16	11	11
	Std. Error	5	3	1	1	4	1
	Fold Change	1	2	1	1	1	1

* Denotes *p* < 0.05 statistical significance compared to 5.5 mM glucose.

**Table 3:** Analysis of MCF7 cell surface lipidome.

Serum Glucose Concentrations	Fold Change			Ratio of PI3P:PI(3,4)P2:PIP3Expression
	PI3P	PI(3,4)P2	PIP3	
5.5 mM	-	-	-	1:1.11:1.17
10 mM	0.80 **	1.2 **	1.1	1:1.20:1.40
25 mM	0.79 **	1.3 **	0.98	1:1.30:1.30
50 mM	0.85 **	1.4 **	0.86 **	1:1.42:1.27

** Denotes *p* < 0.005 statistical significance compared to 5.5 mM glucose.

**Table 4:** Statistical analysis of whole cell lipidome in SKBR3 cell line.

Serum Glucose Concentration		PI3P	PI4P	PI(3,4)P2	PI(3,5)P2	PI(4,5)P2	PIP3
**5.5 mM**	Average %	23	19	14	16	8	20
	Std Error	4	1	2	2	1	3
**10 mM**	Average %	35	14 *	12	14	8	18
	Std Error	4	1	2	2	1	3
	Fold Change	2	1	1	1	1	1
**25 mM**	Average %	31	12 *	13	15	9	20
	Std Error	4	1	2	2	1	3
	Fold Change	1	1	1	1	1	1
**50 mM**	Average %	31	12	13	15	9	19
	Std Error	4	1	2	2	1	3
	Fold Change	1	1	1	1	1	1

* Denotes *p* < 0.05 statistical significance compared to 5.5 mM glucose.

**Table 5:** Analysis of SKBR3 cell surface lipidome.

Serum Glucose Concentrations	Fold Change			Ratio of PI3P:PI(3,4)P2:PIP3 Expression
	PI3P	PI(3,4)P2	PIP3	
5.5 mM	-	-	-	1:0.98:0.65
10 mM	0.84 **	0.99	1.25 **	1:1.10:0.97
25 mM	0.93 **	1.04	1.04	1:1.10:0.73
50 mM	0.87 **	0.98	1.22 **	1:1.10:0.92

** denotes *p* < 0.005 statistical significance compared to 5.5 mM glucose.

**Table 6:** Statistical analysis of whole cell lipidome in MDA-MB-468 Cell Line.

Serum Glucose Concentration		PI3P	PI4P	PI(3,4)P2	PI(3,5)P2	PI(4,5)P2	PIP3
**5.5 mM**	Average %	25	13	15	16	9	22
	Std Error	3	2	2	1	3	1
**10 mM**	Average %	23	14	15	16	9	23
	Std Error	3	2	2	1	3	1
	Fold Change	1	1	1	1	1	1
**25 mM**	Average %	23	11	17	17	11	21
	Std Error	3	2	2	1	3	1
	Fold Change	1	1	1	1	1	1
**50 mM**	Average %	17	11	19	19	14	20
	Std Error	3	2	2	1	3	1
	Fold Change	1	1	1	1	2	1

**Table 7:** Analysis of TNBC cell surface lipidome.

Serum Glucose Concentrations	Fold Change			Ratio of PI3P:PI(3,4)P2:PIP3 Expression
	PI3P	PI(3,4)P2	PIP3	
5.5 mM	-	-	-	1:0.73:0.30
10 mM	0.76 **	1.09 **	1.62 **	1:1.05:0.65
25 mM	0.71 **	1.07	1.79 **	1:1.09:0.75
50 mM	0.61 **	1.02	2.23 **	1:1.21:1.09

** Denotes *p* < 0.005 statistical significance compared to 5.5 mM glucose.

**Table 8:** Overview of PI lipids and associated tumorigenic processes.

Lipid	Cellular Localization	Attributed Process
PI3P	Apical membranes and microdomains of early endosome [43,81,82,83,84,85,86]	Proliferation
PI4P	Golgi apparatus [51,87,88,89,90]	Motility
PI(3,4)P2	Plasma membrane apical membrane focal adhesions [91,92,93]	Motility
PI(4,5)P2	Plasma Membrane apical polarity complexes, Invadopodia [30,87,88,94,95,96,97,98,99]	Motility
PI(3,5)P2	Cytoplasm [100]	Motility
PIP3	Cytoplasm and basolateral membranes [27,101,102,103]	Proliferation and motility

## Abbreviations

MDPI	Multidisciplinary Digital Publishing Institute
DOAJ	Directory of open access journals
TLA	Three letter acronym
EGFR	Epidermal growth factor receptor
ER	Estrogen receptor
PR	Progesterone receptor
HR	Hormone receptor
HER	Human epidermal growth factor receptor
IDC	Invasive ductal carcinoma
PI	Phosphatidylinositol
PI3P	Phosphatidylinositol 3-phosphate
PI4P	Phosphatidylinositol 4-phosphate
PI5P	Phosphatidylinositol 5-phosphate
PI(3,4)P2	Phosphatidylinositol 3,4-bisphosphate
PI(3,5)P2	Phosphatidylinositol 3,5-bisphosphate
PI(4,5)P2	Phosphatidylinositol 4,5-bisphosphate
PIP3	Phosphatidylinositol 3,4,5-triphosphate
TNBC	Triple negative breast cancer
